# Trends in incidence of atopic disorders in children and adolescents - Analysis of German claims data

**DOI:** 10.1016/j.waojou.2023.100797

**Published:** 2023-07-05

**Authors:** Claudia Kohring, Manas K. Akmatov, Lotte Dammertz, Joachim Heuer, Jörg Bätzing, Jakob Holstiege

**Affiliations:** Department of Epidemiology and Health Care Atlas, Central Research Institute of Ambulatory Health Care in the Federal Republic of Germany, Salzufer 8, 10587, Berlin, Germany

**Keywords:** Allergy, Asthma, Atopic dermatitis, Cumulative incidence, Hay fever

## Abstract

**Background:**

This claims-based study aimed to assess recent nationwide trends in pediatric incidence of atopic diseases in Germany.

**Methods:**

Incidence of atopic dermatitis, asthma, and hay fever was assessed from 2013 to 2021 in annual cohorts of 0- to 17-year-old children and adolescents with statutory health insurance (N = 11,828,525 in 2021).

**Results:**

Incidence of atopic dermatitis remained largely unchanged (15.2 cases per 1000 children in 2021) while hay fever incidence exhibited a fluctuating trend over the study period and amounted to 8.8 cases per 1000 in 2021. Asthma incidence decreased gradually between 2013 (12.4/1000) and 2019 (8.9/1000). This downward trend was followed by a further disproportionate reduction from 2019 to 2020 (6.3/1000) and a re-increase in 2021 (7.2/1000).

**Conclusion:**

The findings complement nationwide prevalence surveys of atopic diseases in children and adolescents in Germany. Knowledge about temporal variations in risk of atopic diseases are crucial for future investigations of explanatory factors to enhance the development of preventive measures. While asthma incidence followed a declining trend throughout the study period, an unprecedentedly strong reduction in pediatric asthma risk was observed in 2020, the first year of the COVID-19-pandemic.

To the Editor,

German cross-sectional studies indicate a stagnating prevalence of asthma and hay fever in 0- to 17-year-olds between the survey periods 2003–2006 and 2014–2017,^1^ while claims-based studies observed slightly decreasing trends.^2^^,^^3^ Studies that investigate recent incidence trends of pediatric atopic diseases in Germany are lacking. We calculated nationwide annual and quarterly incidence of atopic dermatitis, hay fever, and asthma in the pediatric population with statutory health insurance (SHI) from 2013 until 2021. In Germany, SHI covered about 84% of the pediatric population in 2021. The occurrence of newly diagnosed atopic diseases was assessed in annual cohorts of patients in the age group 0–17 years, who were observable in the year of reporting and in the 3 previous years or who were born in this four-year period. Incident cases were identified using diagnoses coded according to the International Statistical Classification of Diseases and Related Health Problems, 10th revision, German modification (ICD-10-GM, atopic dermatitis: L20, hay fever: J30.1, J30.2, asthma: J45). For SHI-physicians, it is mandatory to additionally assign a modifier indicating the diagnostic certainty, whereby only diagnoses marked as “assured” were included. This does not provide any information about the diagnostics used, allergens or disease severity in each individual case. A detailed description of applied methods is provided in the online supplemental material.

The annual incidence of atopic dermatitis was stable over time and amounted to 15.2 cases per 1000 persons in 2021 (Fig. 1). Regarding seasonality, the lowest incidence of atopic dermatitis was observed in the third quarter (July to September) and the highest in the first quarter (January to March), varying on an annual average by a factor of 1.8. The peak of new-onset atopic dermatitis in winter months likely results from a generally reduced skin barrier function and increased susceptibility towards mechanical stress due to relatively low humidity and temperature.^4^ The stagnating annual incidence trend is in accordance with findings for Danish and Swedish children for the study periods 1997–2011 and 2006–2010, respectively.^5^ In contrast, hay fever incidence declined by 23% from 2013 to 2017 (8.6 vs 6.6/1000) and re-increased by 33% until 2021 (8.8/1,000, Fig. 1). Specific seasonality was observed for all 3 allergic conditions with hay fever incidence displaying the strongest seasonal variations (Fig. 2). In all years, hay fever incidence was lowest in the fourth and highest in the second quarter, varying on average by factor 3.8. The second calendar quarter comprises the months of April to June and coincides with the period with the highest grass pollen count in Germany.^6^ Although seasonal patterns of hay fever incidence are primarily determined by pollen frequency, fluctuations of the annual cumulative incidence are less well understood. The extent to which the observed changes in annual hay fever incidence can be explained by annual variations in pollen counts requires further investigation, particularly in the context of global warming.

**Fig. 1:**
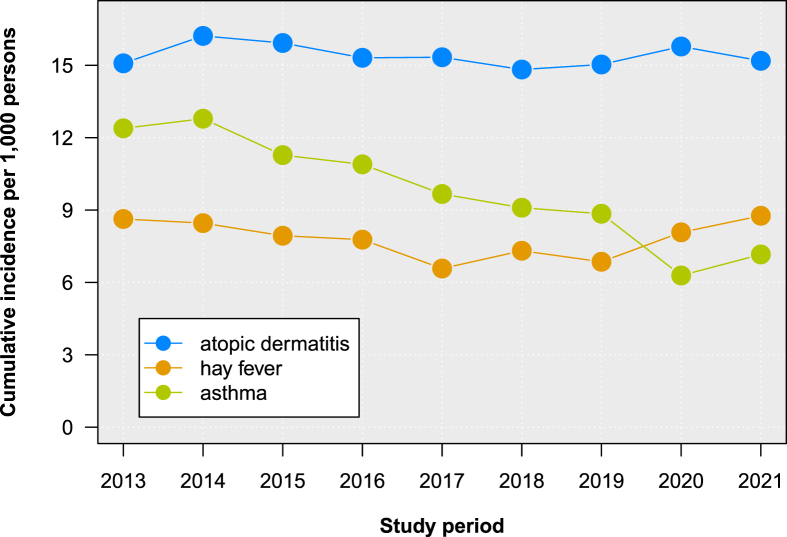
Annual cumulative incidence of atopic dermatitis, hay fever and asthma in children and adolescents (0–17 years) from 2013 to 2021 (per 1000 persons) in Germany

**Fig. 2:**
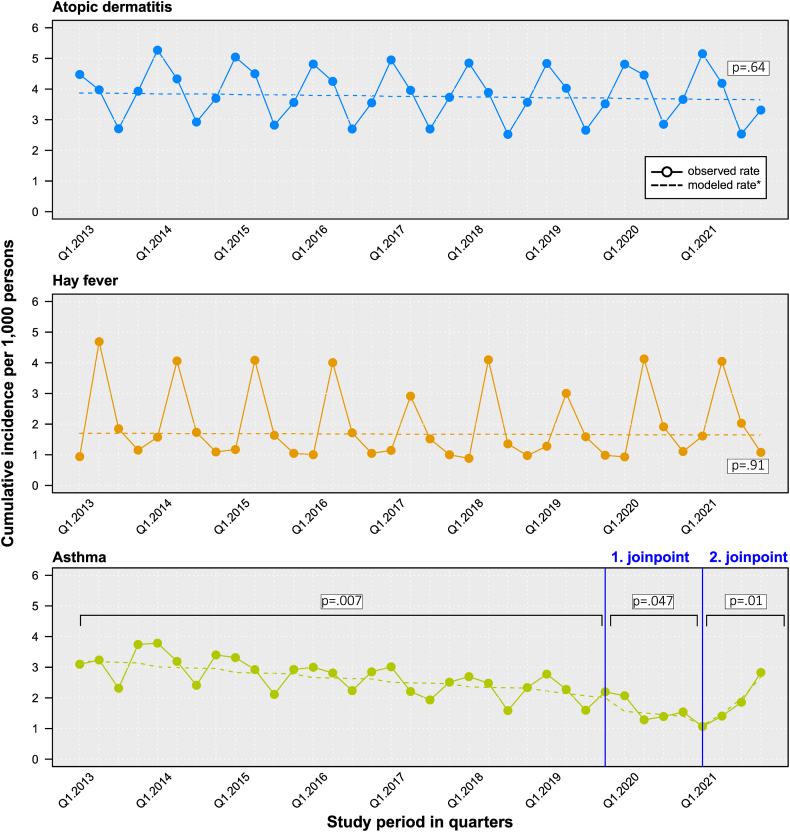
Quarterly cumulative incidence of atopic dermatitis, hay fever, and asthma in children and adolescents (0–17 years) from 2013 to 2021 (per 1000 persons) in Germany. ∗ Modeled incidence rates were estimated with joinpoint regression analysis. We observed no evidence of joinpoints for atopic dermatitis (p = 0.64) and hay fever (p = 0.91), but 2 joinpoints for asthma (blue vertical lines). The trends in all 3 periods observed (Q1.2013-Q3.2019, Q4.2019-Q4.2020, and Q1-Q4.2021) were statistically significant

Between 2013 and 2019, asthma incidence exhibited an almost constant decrease from 12.4 to 8.9 cases per 1000 persons, followed by a further disproportionately strong reduction in 2020 (6.3/1,000, Fig. 1). In 2021, annual incidence increased again to 7.2 cases per 1000 persons. The quarterly relative difference between the highest and lowest values within a year varied by factor 1.6. In the pre-pandemic period (2013–2019), the lowest asthma incidence was observed in the third quarter (July to September) each year. This seasonal pattern, observed for years pre-pandemically, was broken in 2020 and 2021 and shows no discernible pattern during this period. While quarterly incidence in the first quarter of 2021 was at an all-time low, in the third quarter of 2021 it exceeded the corresponding values in 2018 and 2019, and in the fourth quarter of 2021 it even exceeded incidence in the fourth quarter of 2017 (Fig. 2). To our knowledge, our study is the first to describe a marked and stepwise decrease of physician-diagnosed asthma in the German pediatric population. Due to the different dynamics over the study course, it appears reasonable to differentiate between trends before and during the SARS-CoV-2 pandemic. In the pre-pandemic time under study (2013–2019), we observed a steady decrease of asthma incidence. Various environmental and behavioral factors associated with asthma may have contributed to the declining trend. These include a strong and contemporaneous decrease of antibiotic use in young children,^7^ for which there reportedly is an association with the risk of childhood asthma.^8^ In Germany, between 2010 and 2018 the antibiotic prescription rate in the age group 0–1 year fell by almost 50% from 630 to 320 prescriptions per 1000 children.^9^ Similar to Germany, reductions in childhood asthma risk were accompanied by decreasing antibiotic use in infancy on the population level in Canada.^8^ Smoking and exposure to tobacco smoke are also known to contribute high population attributable risk for asthma.^10^ Teenagers without a previous history of asthmatic symptoms who started smoking had an increased risk for asthma later in life.^11^ Furthermore, exposure to passive smoking in childhood is positively associated with asthma and children whose mothers smoked during pregnancy exhibit an increased asthma risk.^12^ In Germany, active smoking behaviour and exposure to passive smoke of children and expecting mothers changed substantially between the years 2003 and 2017 and might explain reductions in the asthma incidence.^13^ From 2019 to 2020, marking the first year of the SARS-CoV-2 pandemic, asthma incidence in our study decreased disproportionately by 29%. Since seasonal incidence patterns of atopic dermatitis and hay fever did not change in 2020 and 2021 and annual hay fever incidence even increased, we suggest this is unlikely to be an artificial effect of lower health care utilization due to SARS-CoV-2 pandemic-related contact restrictions. This notion is supported by the finding that contrary to the pre-pandemic period, the annual peak in the fourth quarter almost disappeared in 2020, but seasonal incidence patterns remained largely unchanged for atopic dermatitis and hay fever. While there is evidence that COVID-19 lockdowns were followed by reductions in asthma exacerbations as well as hospitalizations due to severe respiratory infections in infants and young children in multiple settings,^14^^,^^15^ our findings indicate that pediatric asthma incidence declined strongly as well. Still, contact restrictions and temporal closing of kindergartens and schools may have been an explanatory factor for the marked decrease in asthma incidence in the first pandemic year.^7^ For 2020, laboratory-based sentinel data indicate a marked decline of circulating respiratory viruses, including rhinovirus (RV) and human respiratory syncytial virus (RSV).^16^ A reduced RV and RSV activity during 2020 may have contributed to the strong decline in asthma incidence in Germany. Nevertheless, a surge in incidence in 2021 may indicate that the onset of asthma was merely postponed in many children. It is essential to prospectively monitor asthma incidence trends in early childhood.

Our study has some limitations. Data are limited to patients with symptomatic disease that required outpatient treatment. The dataset does not contain clinical information (eg, specific allergens, severity of symptoms/disease, diagnostic tests performed), inpatient data, or family history of atopic disease. As data availability was limited to 10 years, we had to select a suitable trade-off between defining a sufficiently long diagnosis-free pre-observation period to avoid misclassification of prevalent cases and assess incidence trends for as many years as possible. Using a 3-year diagnosis-free pre-observation period, we provide recent incidence trends of atopic disorders in the German pediatric population over an extended period of 9 years. Our findings complement nationwide cross-sectional, interview-based surveys of atopy prevalence in children and adolescents in Germany.^1^ Knowledge about temporal variations of population risk may allow to narrow down possible risk factors, eg, environmental exposure, and inform preventive measures.

## Abbreviations

COVID-19, coronavirus disease 2019; SARS-CoV-2, severe acute respiratory syndrome coronavirus 2; SHI, statutory health insurance; RSV, respiratory syncytial virus; RV, rhinovirus.

## Funding statement

No funding was received for this study.

## Availability of data and materials

The data analyzed in this study are not publicly available due to the data protection regulations of the German Social Code Book (Fünftes Sozialgesetzbuch, SGB V).

## Author contributions

JHo conceived the study and was primarily responsible for study design and data analysis. CK was involved in further developing the study design and statistical analysis and wrote the manuscript's first draft. MKA was involved in data analysis and visualised the results. LD and JHe were involved in further developing the manuscript. MKA, LD, JHe, JHo and JB critically reviewed the manuscript and were involved in interpretation of results. JB and JHo supervised the project. All authors read and approved the final version of the manuscript and gave final consent for publication.

## Ethical statement

The use of claims data for scientific research is regulated by the Code of Social Law (SGB V) in Germany. An ethical approval and informed consent are not required as this study used routinely collected anonymized data. The research was conducted in accordance with the Helsinki Declaration (in its current revised form: 64th WMA General Assembly, Fortaleza, Brazil, October 2013).

## Submission declaration

All authors have approved the submission of this manuscript. The results have not been previously published and are not being considered for publication in another journal.

## Declaration of competing interest

The authors reported no financial interests or potential conflicts of interest related to this study.
