# Supplementary material for: Trends in incidence of atopic disorders in children and adolescents - Analysis of German claims data
Source: World Allergy Organ J. 2023 Jul 5;16(7):100797. doi: 10.1016/j.waojou.2023.100797 (PMC10359926; doi:10.1016/j.waojou.2023.100797)
Supplement: Multimedia component 1 [file mmc1.pdf]

Material and methods

Data and study population

This study is based on outpatient claims data of the German Statutory Health Insurance (SHI) collected according to §295 of the German Social Code Book V (Sozialgesetzbuch V, SGB V) from the year 2013 until the third calendar quarter of 2022, the latest quarter of data availability. The data comprise information for the SHI insured population in Germany who had at least one consultation to a SHI-accredited physician in the respective year. In 2021, SHI covered about 88% of the total German population [1] and about 84% of the age group 0-17 years [2,3].

The study's source population consisted of all children and adolescents aged 0 to 17 years (N=11,828,525 in 2021). Available information include sociodemographic data (e.g. age, sex, residential region), diagnoses, medical services for insurees and physician-related characteristics (e.g. specialisation, practice information). Diagnoses are coded in accordance with the International Statistical Classification of Diseases and Related Health Problems, 10th revision, German modification (ICD-10-GM). For SHI-physicians, it is mandatory to additionally assign a disease modifier that specifies the diagnostic certainty as either "assured", "suspected", "status post" or "excluded". The atopic diseases of interest were atopic dermatitis (ICD-10-code: L20), hay fever (J30.1, J30.2) and asthma (J45).

The occurrence of newly diagnosed atopic diseases was assessed in annual cohorts of patients in the age group 0-17 years, who were observable in the year of reporting and in the three previous years or who were born in this four-year period. We assumed a pre-observational period of three years as fulfilled for patients with an outpatient visit in the year of reporting, if there was at least one outpatient visit in the calendar year three years before the reporting year or earlier. For children born in the three-year pre-observation period or in the reporting year, at least one outpatient visit in the year of birth and one in the year of reporting was sufficient for study inclusion. This includes children who were born in the respective year of reporting.

Population at risk and case definition

Subpopulations at risk for the occurrence of atopic dermatitis, hay fever and asthma were independently extracted from the annual cohort populations. All patients who did not receive a diagnosis of atopic dermatitis, hay fever or asthma, respectively, during the pre-observation period were eligible for inclusion into the disease-specific population at risk (figure S1). New disease cases of atopic dermatitis, hay fever or asthma were defined as the first occurrence of disease-specific diagnostic codes in the respective subpopulations at risk, together with the disease modifier "assured" after a diagnosis-free period of three years and repeated coding at least once in the following four

quarters after the index quarter. For instance, a first diagnosis of asthma in the 1st quarter of 2013 (index quarter) needed at least one confirmatory asthma diagnosis in the following period from 2nd quarter/2013 to 1st/2014. We chose this approach with respect to the seasonality in the occurrence of atopic diseases, as an initial diagnosis could be verified by a recurring diagnosis in a time window that included the same quarterly season in the following year.

Statistical analysis

We calculated the annual cumulative incidence for the studied atopic diseases per 1,000 insured children and adolescents under risk for the years 2013 to 2021. Disease-specific results are stratified by sex and age groups (0-2, 3-6, 7-10 and 11-17 years). Seasonality of trends was assessed based on the quarterly cumulative incidence (1st/2013-4th/2021).

For first diagnoses documented in the 4th quarter of 2021, there was no sufficient follow-up time for the observation of confirmatory diagnoses coded in the following four quarters as required by the case definition. Therefore, we extrapolated the case numbers of the three diseases under study for the 4th quarter of 2021 by calculating disease-specific positive predictive values (ppv) based on the study period with sufficient follow-up for the confirmation of initial diagnoses. We calculated ppv as the arithmetically averaged percentage of first diagnoses in the fourth calendar quarter in the period from 2013 to 2020 that were confirmed in the respective following four quarters. We used age group- and sex-specific ppv for each disease under study to extrapolate incident cases according to the case definition.

Temporal trends in the incidence of the three atopic conditions were analysed via joinpoint regression model. Briefly, joinpoint regression fits the simplest model to the input data by starting with the minimum number of joinpoints and tests the model fit up to the maximum (min=0, max=5). For each iteration, the program tests whether increasing the number of joinpoints leads to a better model fit. This analysis was performed with the program *Joinpoint* (version 4.9.0.0), developed by the National Cancer Institute in the USA [4].

References „Material and methods“

1. Verband der Ersatzkassen e. V. (vdek). vdek-Basisdaten des Gesundheitswesens in Deutschland 2022. 2022. https://www.vdek.com/presse/daten/_jcr_content/par/publicationelement_1479644990/file.res/VDEK_Basisdaten2022_210x280_RZ-X4_ohne_Schnittmarken.pdf. Accessed 20.09.2022.
2. Statistisches Bundesamt (Destatis). GENESIS-Tabelle: 12411-0005, Bevölkerung: Deutschland, Stichtag, Altersjahre, Fortschreibung des Bevölkerungsstandes Deutschland. 2021; https://www-genesis.destatis.de/genesis/online. Accessed 03.11.2021.
3. Bundesministerium für Gesundheit (BMG). Statistik über Versicherte, gegliedert nach Status, Alter, Wohnort und Kassenart, Stichtag: 1. Juli 2021 (KM6-Mitgliederstatistik). In. Bonn/Berlin2021.
4. National Cancer Institute. Joinpoint Regression Program, Version 4.9.0.0 - March 2021; Statistical Methodology and Applications Branch, Surveillance Research Program. 2021.

doi: https://doi.org/10.1016/j.waojou.2023.100797

Figures and additional findings

Reporting year	2013	2014	2015	2016	2017	2018	2019	2020	2021
**Source population**	N=11,277,529	N=11,224,970	N=11,239,008	N=11,441,595	N=11,627,879	N=11,810,559	N=11,761,061	N=11,735,928	N=11,828,525
**Cohort population**	n=10,198,866	n=10,193,000	n=10,205,209	n=10,275,639	n=10,329,263	n=10,460,593	n=10,590,137	n=10,716,073	n=10,877,204
**Population at risk**									
AD	n=8,589,133	n=8,623,341	n=8,644,173	n=8,714,078	n=8,771,905	n=8,902,313	n=9,035,033	n= 9,162,140	n= 9,309,272
HF	n=9,515,518	n=9,509,047	n=9,525,463	n=9,600,101	n=9,664,639	n=9,823,658	n=9,959,610	n=10,102,877	n=10,251,672
AST	n=9,059,254	n=9,065,304	n=9,069,918	n=9,144,391	n=9,211,770	n=9,376,279	n=9,540,312	n= 9,703,635	n= 9,936,783
**Incident cases**									
AD	n=129,547	n=139,855	n=137,659	n=133,426	n=134,501	n=131,974	n=135,827	n=144,573	n=141,363
HF	n= 82,107	n= 80,431	n= 75,583	n= 74,626	n= 63,554	n= 71,838	n= 68,280	n= 81,585	n= 89,837
AST	n=112,221	n=115,914	n=102,284	n= 99,625	n= 89,033	n= 85,281	n= 84,388	n= 61,014	n= 71,143

Figure S1. Flow chart depicting the different populations that gave rise to the cases for all reporting years. The source population comprises all statutory health insured children and adolescents aged 0-17 years in Germany.
AD - atopic dermatitis, HF - hay fever, AST - asthma

Age- and sex -specific incidence trends

For atopic dermatitis, by far the highest incidence was observed in the age group 0-2 years (girls: 40/1,000 persons, boys: 49/1,000 in 2021, figure S2). Only in this age group, the incidence in 2021 exceeded the incidence in 2013, the first year of observation, while the remaining age groups displayed a moderately declining trend. Regarding sex differences, except for the youngest age group, girls were more affected (figure S2).

For hay fever, 2017 showed the lowest incidence over the study period in boys and girls and almost all age groups, except for 0-2 year old children (figure S2). Whilst the general trend was similar for females and males, the overall increase in incidence between 2017 and 2021 was more pronounced in the latter. Children aged 0-2 years showed the lowest incidence throughout the study period. In almost all age groups, the incidence was higher in males than females (figure S2).

In contrast to atopic dermatitis and hay fever, a clear downward trend in asthma incidence was observed throughout the study period for both, girls and boys of all ages (figure S2). Regardless of sex, the highest incidence was noticed in the youngest age group (0-2 years). Only in the age groups 0-2 and 3-6 years incidence showed a disproportionately strong reduction from 2019 to 2020 and a re-increase in 2021, without reaching its level in 2019. Among boys, the incidence decreased with advancing age. Among girls, the lowest asthma incidence was observed at the age of 7-10 years with a renewed increase in the older age group in all reporting years (figure S2).

doi: https://doi.org/10.1016/j.waojou.2023.100797

Our findings regarding age- and sex-specific differences are in accordance with other studies examining the incidence of atopic diseases [1]. The causes of this observation can possibly be explained by hormonal factors that differ between the sexes and change with increasing age [2].

References “Age- and sex -specific incidence trends”

1. Henriksen L, Simonsen J, Haerskjold A, Linder M, Kieler H, Thomsen SF et al. Incidence rates of atopic dermatitis, asthma, and allergic rhinoconjunctivitis in Danish and Swedish children. J Allergy Clin Immunol. 2015; doi: 10.1016/j.jaci.2015.02.003.
2. Chen, W., Mempel, M., Schober, W., Behrendt, H. and Ring, J. (2008), Gender difference, sex hormones, and immediate type hypersensitivity reactions. Allergy, 63: 1418-1427. https://doi.org/10.1111/j.1398-9995.2008.01880.x

doi: https://doi.org/10.1016/j.waojou.2023.100797

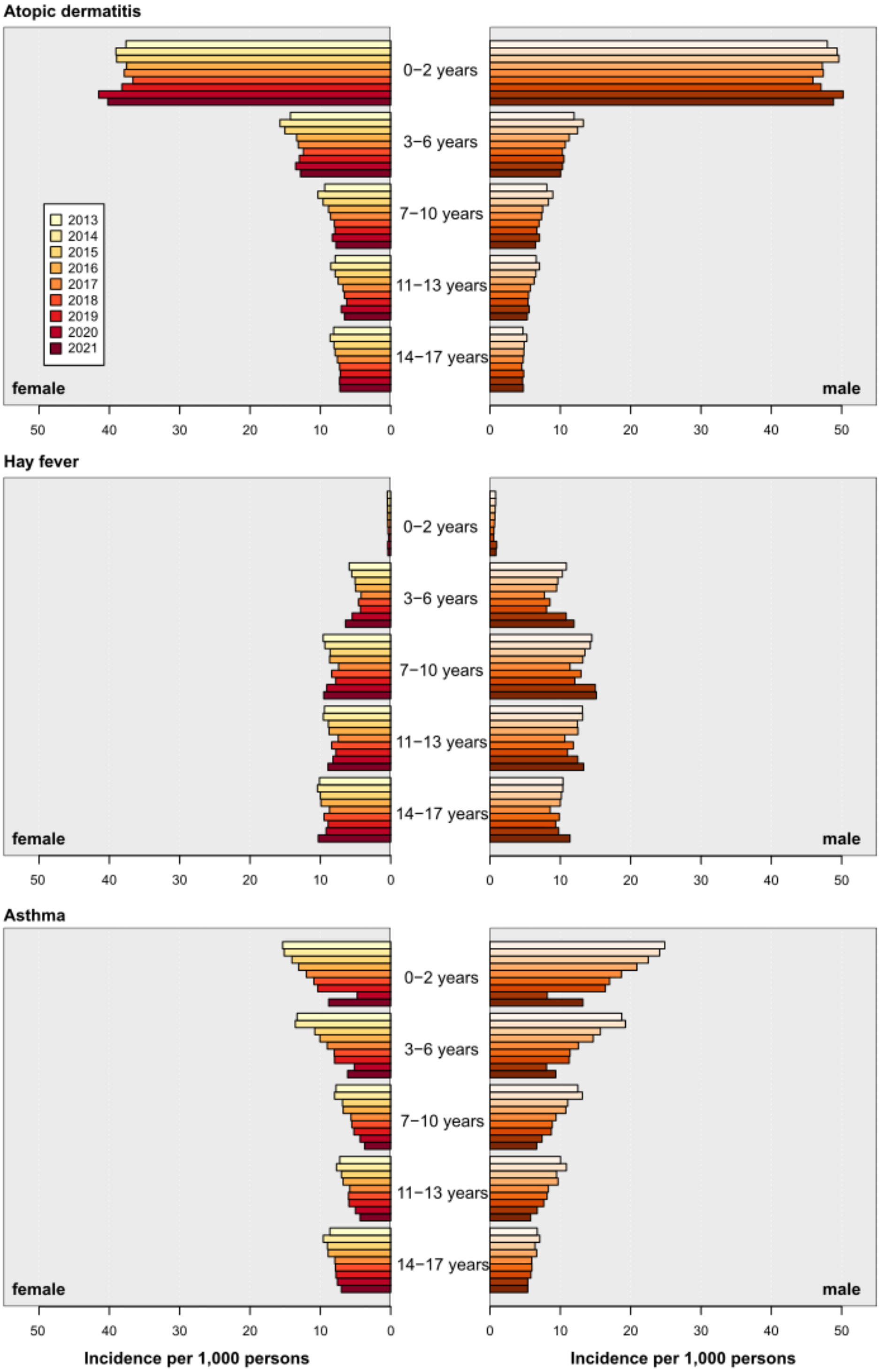

Figure S2. Annual age- and sex-specific cumulative incidence of atopic dermatitis, hay fever and asthma in the period from 2013 to 2021 in the paediatric population with statutory health insurance aged 0-17 years in Germany.
